# Supplementary material for: The interplay between peptides and RNA is critical for protoribosome compartmentalization and stability
Source: Nucleic Acids Res. 2024 Sep 28;52(20):12689–700. doi: 10.1093/nar/gkae823 (PMC11551759; doi:10.1093/nar/gkae823)
Supplement: gkae823_Supplemental_File [file gkae823_supplemental_file.pdf]

SUPPLEMENTARY INFORMATION

The interplay between peptides and RNA is critical for protoribosome compartmentalization and stability

Simone Codispoti[1†], Tomoko Yamaguchi[2,3†$], Mikhail Makarov[3], Valerio G. Giacobelli[3], Martin Mašek[4], Michal H. Kolář[4], Alma Carolina Sanchez Rocha[3], Kosuke Fujishima[2,5,6*], Giuliano Zanchetta[1*], Klára Hlouchová[3,7*]

[1]*Dipartimento di Biotecnologie Mediche e Medicina Traslazionale, Università di Milano, Segrate, 20054, Italy*

[2]*Earth-Life Science Institute, Tokyo Institute of Technology, Ookayama, Meguro-ku, Tokyo 152-8550, Japan*

[3]*Department of Cell Biology, Faculty of Science, Charles University, BIOCEV, Prague, 12843, Czech Republic*

[4]*Department of Physical Chemistry, University of Chemistry and Technology, Technicka 5, 16628 Prague, Czech Republic*

[5]*School of Life Science and Technology, Tokyo Institute of Technology, Meguro-ku, Tokyo 152-8550, Japan*

[6]*Graduate School of Media and Governance, Keio University, Fujisawa, 252-0882, Japan*

[7]*Institute of Organic Chemistry and Biochemistry, Czech Academy of Sciences, Prague, 16610, Czech Republic*

[†]These authors contributed equally to this work

[$]Current address: Department of Structural Biology, Graduate School of Pharmaceutical Sciences, Kyoto University, Kyoto, Japan

[*]To whom correspondence should be addressed: fuji@elsi.jp, giuliano.zanchetta@unimi.it, klara.hlouchova@natur.cuni.cz

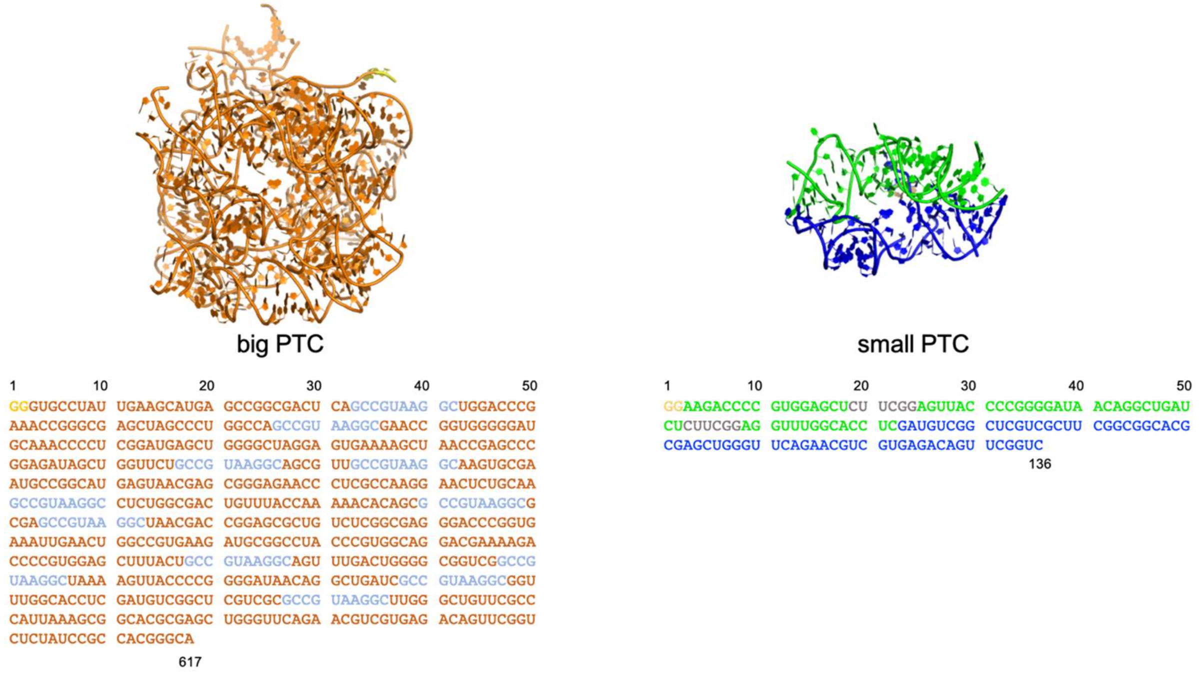

Supplementary Fig. 1. Tertiary structures derived from PDB 4V51 and related sequences of the two WT PTC constructs employed in this study: the 617 nt bPTC (*left panel*) and the 136 nt sPTC (*right panel*). WT bPTC is made up of the 505 nt from the 23S rRNA (*orange*), joined by 11 stem loops (*light blue*). The WT sPTC is composed of 116 nt from the 23S rRNA (A region in *green* & P region in *blue*) and connected with 3 stem loops (*grey*). The extra GG for transcription is marked in *yellow*.

Supplementary Table 1. DNA oligos used for preparation of PTC RNA constructs.

Name	Sequence	Note	Reference
WT_bPTC	AAAAAGTGGCCTGTAATACGACTCACTATAGGGTGCCTATTGAAGCATGAGCCGGCGACTCAGCCGTAAGGCTGGACCCGAAACCGGGCGAGCTAGCCCTGGCCAGCCGTAAGGCGAACCGGTGGGGGATGCAAACCCCTCGGATGAGCTGGGGCTAGGAGTGAAAAGCTAACCGAGCCCGGAGATAGCTGGTTCTGCCGTAAGGCAGCGTTGCCGTAAGGCAAGTGCGAATGCCGGCATGAGTAACGAGCGGGAGAACCCTCGCCAAGGAACTCTGCAAGCCGTAAGGCCTCTGGCGACTGTTTACCAAAAACACAGCGCCGTAAGGCGCGAGCCGTAAGGCTAACGACCGGAGCGCTGTCTCGGCGAGGGACCCGGTGAAATTGAACTGGCCGTGAAGATGCGGCCTACCCGTGGCAGGACGAAAAGACCCCGTGGAGCTTTACTGCCGTAAGGCAGTTTGACTGGGGCGGTCGGCCGTAAGGCTAAAAGTTACCCCGGGGATAACAGGCTGATCGCCGTAAGGCGGTTTGGCACCTCGATGTCGGCTCGTCGCGCCGTAAGGCTTGGGCTGTTCGCCCATTAAAGCGGCACGCGAGCTGGGTTCAGAACGTCGTGAGACAGTTCGGTCTCTATCCGCCACGGGCA	Template of WT bPTC	(1)
Sh_bPTC	AAAAAGTGGCCTGTAATACGACTCACTATAGGCCTCGGCGGACCCACCCCAACCTCGAGTAGGGGACCTACACAGAGCATTATCAAACGACAGCCTTCTCAGGTGCATTAAGGACACCAAAAGATCCAGCCGCCGGATGGGATGAGTGACCGACCGCTACTAGGCCGCATGAGACCCCAATATCCCGTGCAGACAGGGAAAGGCACAGACGAGGTCAGTCAAACTGGTCCGCTTCACAGGGGGAGTCACTGCAAGGTCGTATACTGCGTGGTTGGGTACCGGTCGGGTCTGCTGACCAGTATGAGGTATTCCCTACTCTAGGGATGACCGAAGGGTGTTAGTTCCCGAGGAGATTCCCGCCCAGGAATGCTAGATGGACGCGGGAGCGTGTAGTCAGGGGTAAGACTTAGACACGGGTCCACGTAGCGGTGGCACTGATAGGTGCGTTATGCGCCGCGACGCTGGGCTGGGCCAGGTGACGTCGAGCAGTGCTGACGGCGAAGGGCGGCGCCGCGGGCGAGGATCTAATCAGGCTTTACTGTGCAGTAGGACGGGGATGGCCCGGGCACGCTATACGGCGACGCCCGCCCACGGTAAGCGGGAGAGATGCGACCATCGAGCAGCCGAGTGGTCTATCCGCCACGGGCA	Template of Sh bPTC	This study

bPTC_F	AAAAAGTGGCCTGTAATACGACTCA	Forward primer for preparing dsDNA of bPTC by using DNA polymerase	This study
bPTC_R	TCTATCCGCCACGGGCA	Reverse primer for preparing dsDNA of bPTC by using DNA polymerase	This study
WT_sPTC_F	GCGTAATACGACTCACTATAGGAAGACCCCGTGGAGCTCTTCGGAGTTACCCCGGGGATAACAGGCTGATCTCTTCGGAGGTTTGGCAC	Forward primer for preparing dsDNA of WT sPTC by using Klenow enzyme	(2)
WT_sPTC_R	GACCGAACTGTCTCACGACGTTCTGAACCCAGCTCGCGTGCCGCCGAAGCGACGAGCCGACATCGAGGTGCCAAACCTCCGAAGAG	Reverse primer for preparing dsDNA of WT sPTC by using Klenow enzyme	(2)
Sh1_sPTC_R	GCAACCATATGACCAGCCACAACGTCCCATGGACGTTGCATTCACATTGGGCCGTGCCACGGGGCCCTACGGGTCCCGATCCGAGAAATGGCCCCGTGGCTATCGACCATAACTCGGTGCCCCGGAGCAGGCCAATCCTATAGTGAGTCGTATTACGC	Template of Sh1 sPTC	This study
Sh2_sPTC_R	CAGACACGCCTGGACGACGACCATGCCAAGTCCCTTACAAGCTTTCAGGCACCCACTACACGCGGGCAATACTGCGATGGCCGCGCTCCTTGTCCGCGGCGACGCACGACACAAGTTCAGGTCTGTTGGGGACCAGCCTATAGTGAGTCGTATTACGC	Template of Sh2 sPTC	This study
T7_F	GCGTAATACGACTCACTATAGG	Forward primer for sPTCs	This study

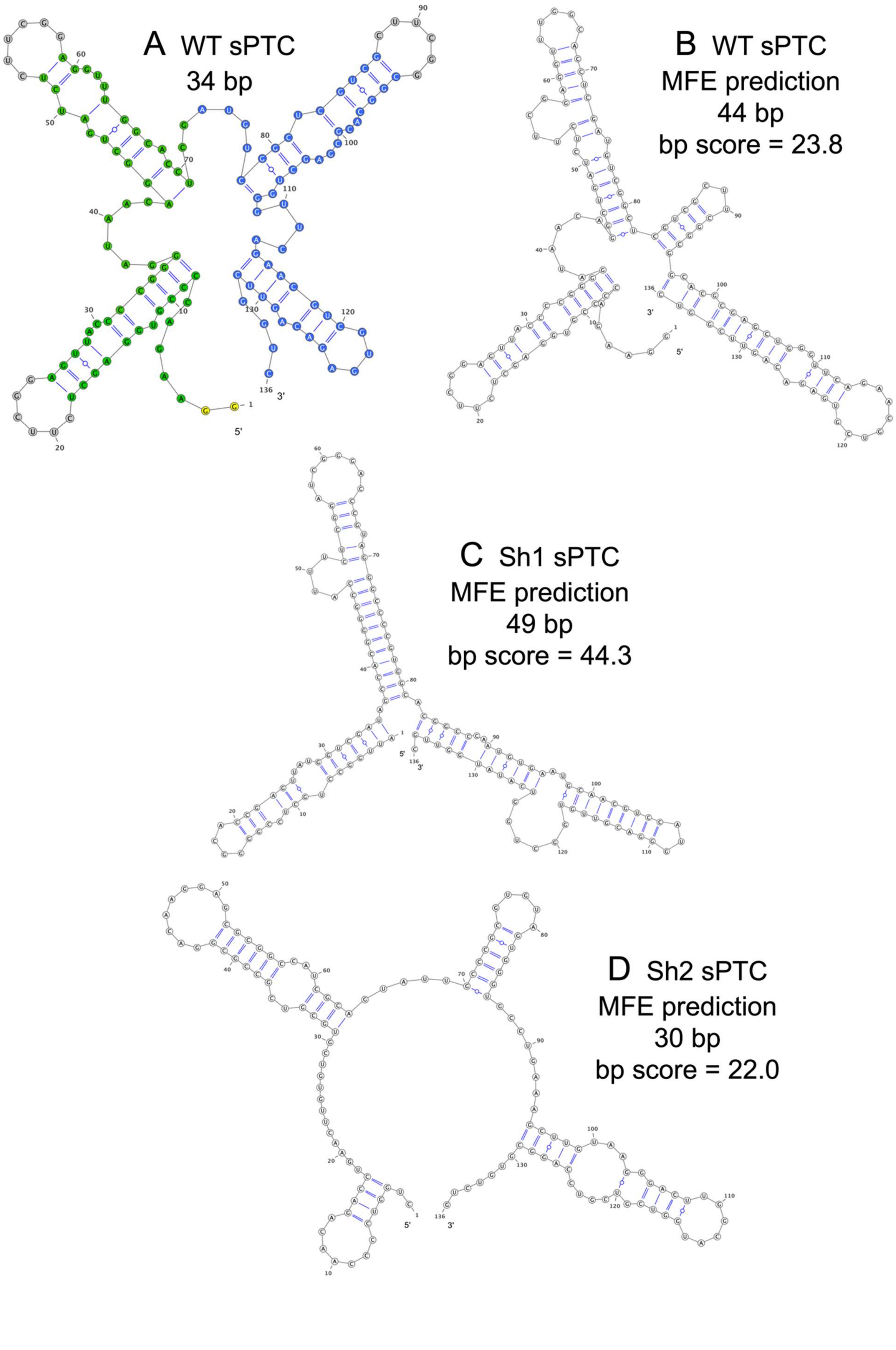
A WT sPTC
34 bp
B WT sPTC
MFE prediction
44 bp
bp score = 23.8
C Sh1 sPTC
MFE prediction
49 bp
bp score = 44.3
D Sh2 sPTC
MFE prediction
30 bp
bp score = 22.0

Supplementary Fig. 2. Secondary structures of WT sPTC and its two shuffled variants.
(A) Original WT sPTC based on PDB 4V51, characterised by 34 total number of base pairs (bp); blue-green colour scheme matches the two symmetrical regions depicted in **Supplementary Fig. 1**, while the additional 3 CUUCGG loops and the starter GG are also inserted and coloured in grey and yellow, respectively.
(B) NUPACK MFE prediction for the WT sPTC, characterised by 44 bp and a “bp score” (see below) of 23.8.
(C - D) NUPACK MFE prediction for the Sh1 and Sh2 sPTC, having respectively more and less structure content compared to the WT sPTC: Sh1 sPTC is characterised by 49 predicted bp and a “bp score” (see below) of 44.3, while Sh2 PTC has 30 predicted bp and a “bp score” of 22.
All predictions (B-D) were performed at the standard 1M NaCl and 37°C conditions adopted by the NUPACK algorithm. The number of base pairs (bp) in the Minimum Free Energy (MFE) is reported, together with a “bp score” computed as the sum of the probabilities that each nucleotide adopts the state of the MFE.

Even if the nominal conditions are different from the ones used in the experiments, it is guaranteed that the two variants assume different conformations, with less and more base pairs at equilibrium with respect to WT sPTC. This is due to the fact that: (i) the predicted structure of the original WT sPTC at 1 M NaCl and 37°C (B) lies in between the structures of Sh1 sPTC and Sh2 sPTC; (ii) monovalent salts and temperature have a monotonic effect on the stability of RNA.

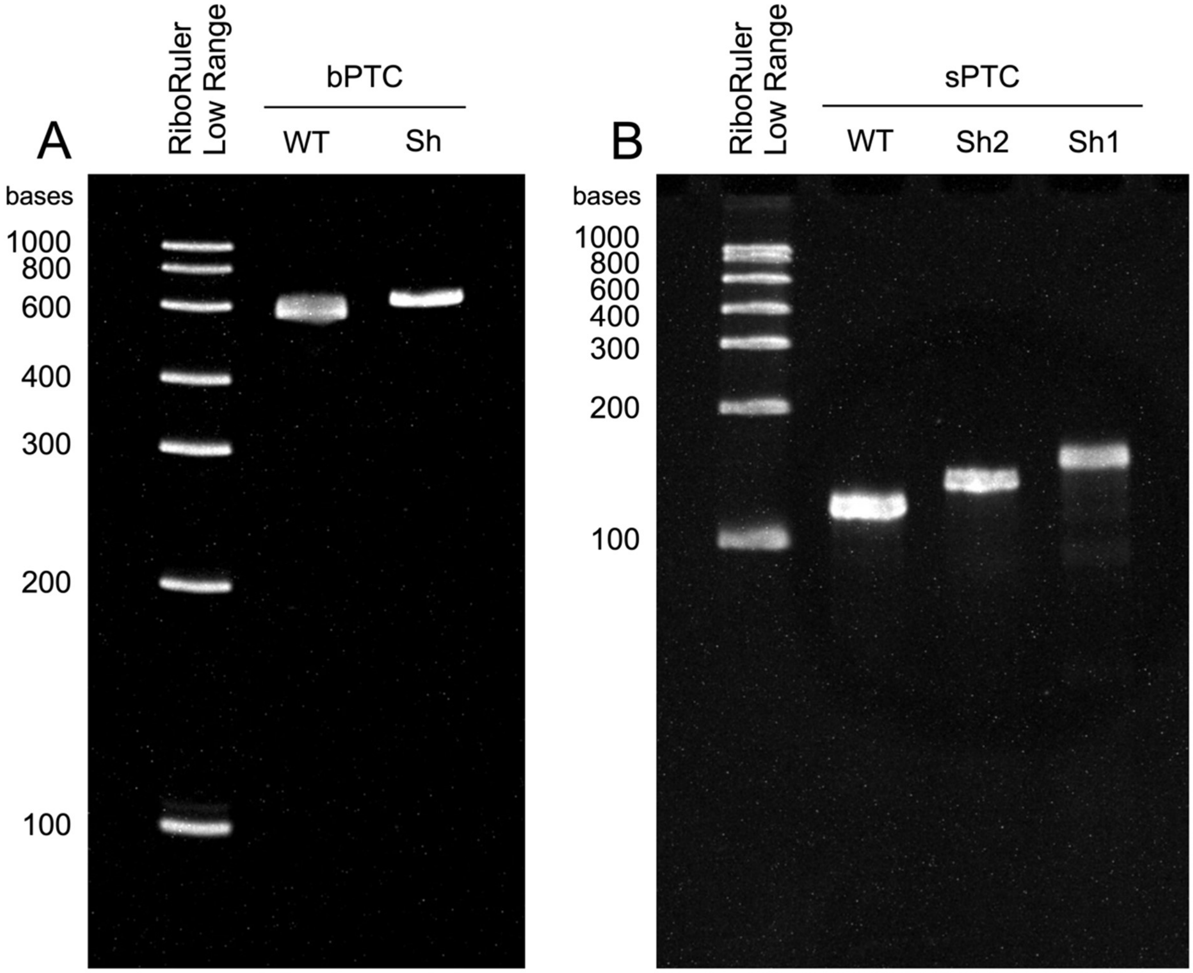

Supplementary Fig. 3. Denaturing gels of the RNA constructs.
(A) 5% TBE-urea gel of WT and Sh bPTC RNA constructs.
(B) 8% TBE-urea gel of WT and Sh sPTC RNA constructs.
Prior to electrophoresis, 200 ng RNA samples were mixed with RNA Gel Loading Dye (2X) (ThermoFisher Scientific) and denatured at 70°C for 10 min. RiboRuler Low Range RNA Ladder (ThermoFisher Scientific) was used as a reference. After electrophoresis, gels were soaked in TBE buffer supplemented with GelRed Nucleic Acid Gel Stain (Sigma Aldrich) for 10 min and visualised by UV transilluminator.

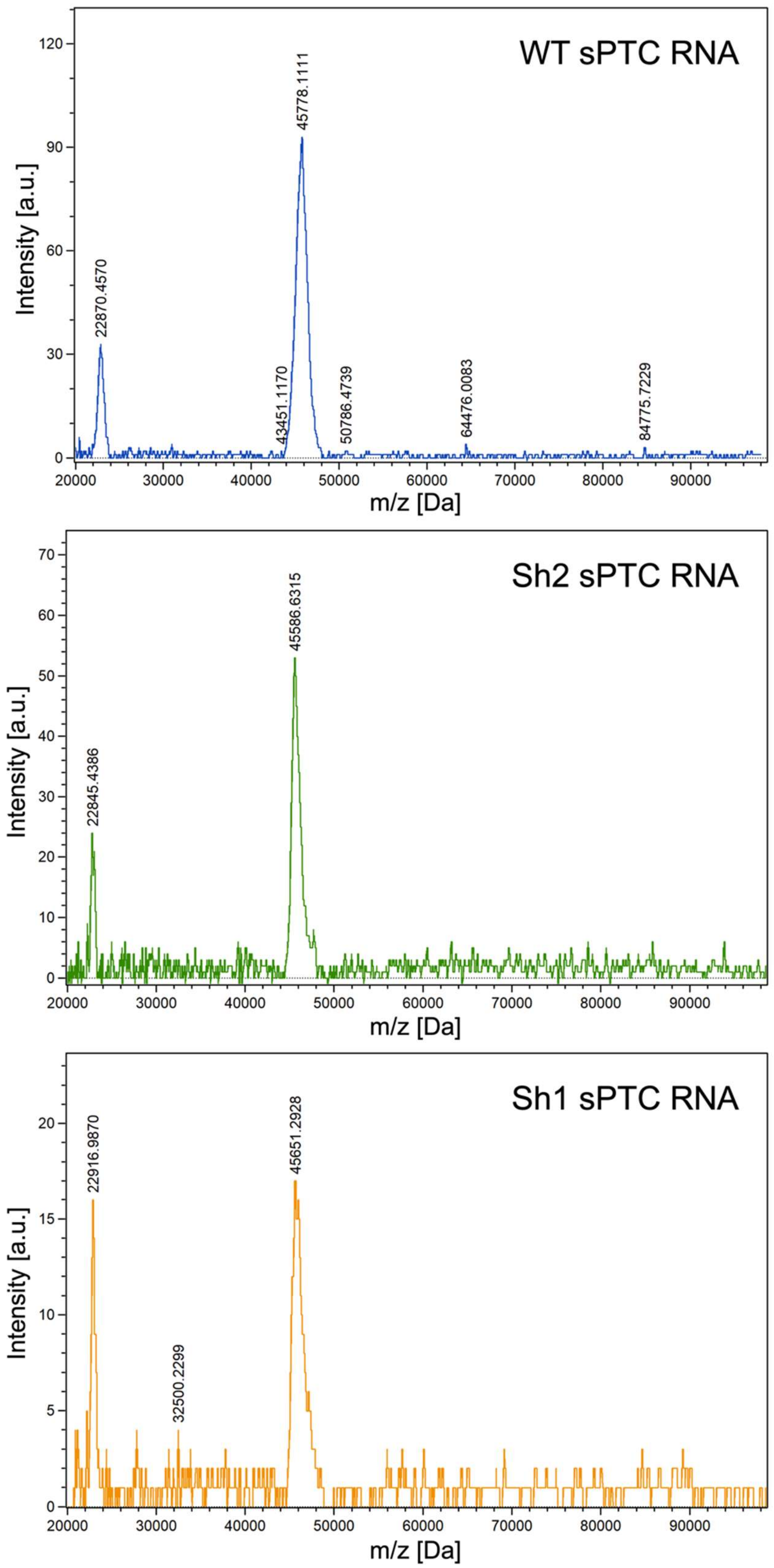

Supplementary Fig. 4. MALDI-TOF spectra of sPTC RNA constructs.

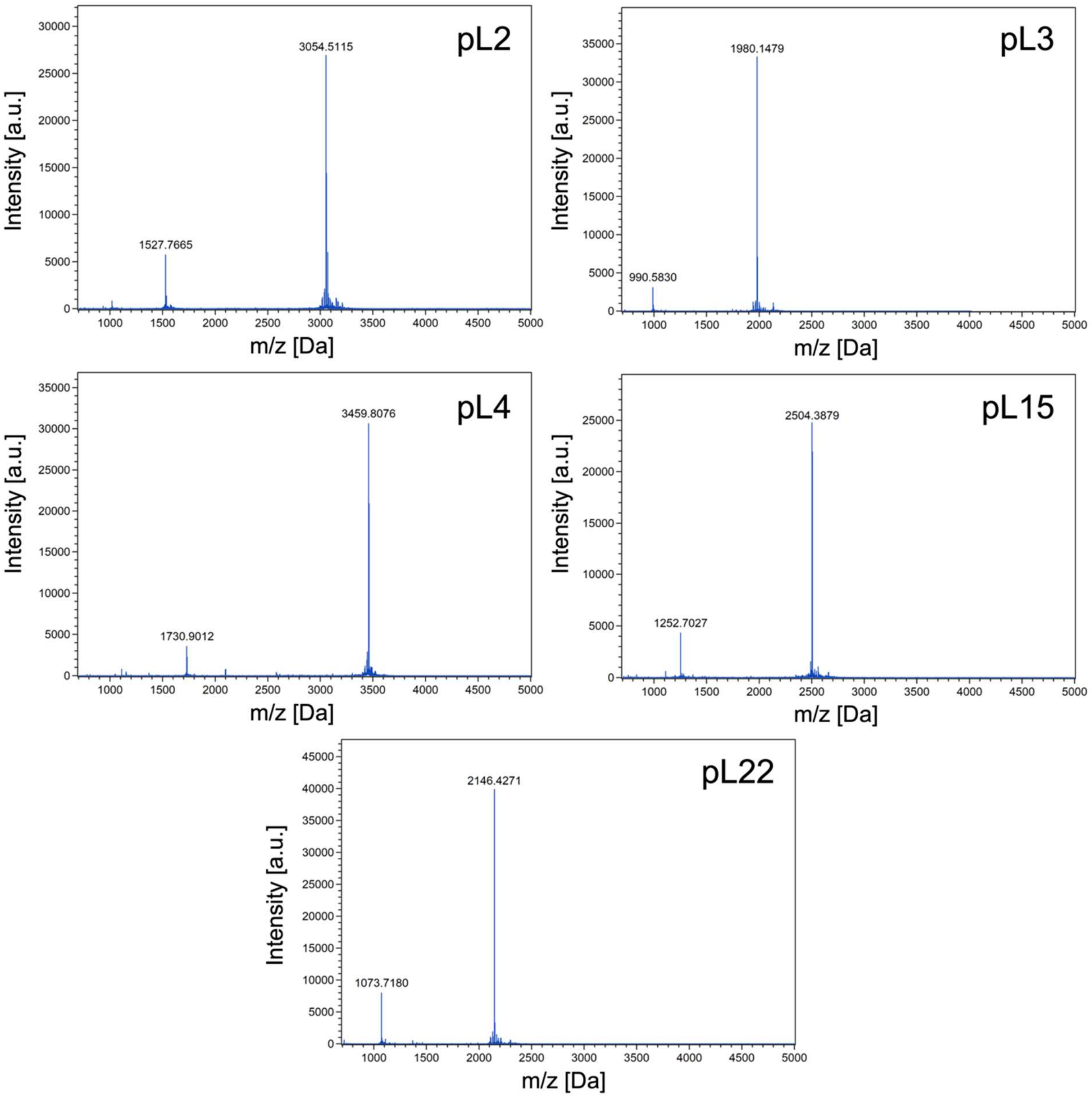

Supplementary Fig. 5. MALDI-TOF spectra of rPeptides (pL2, 3, 4, 15, 22).

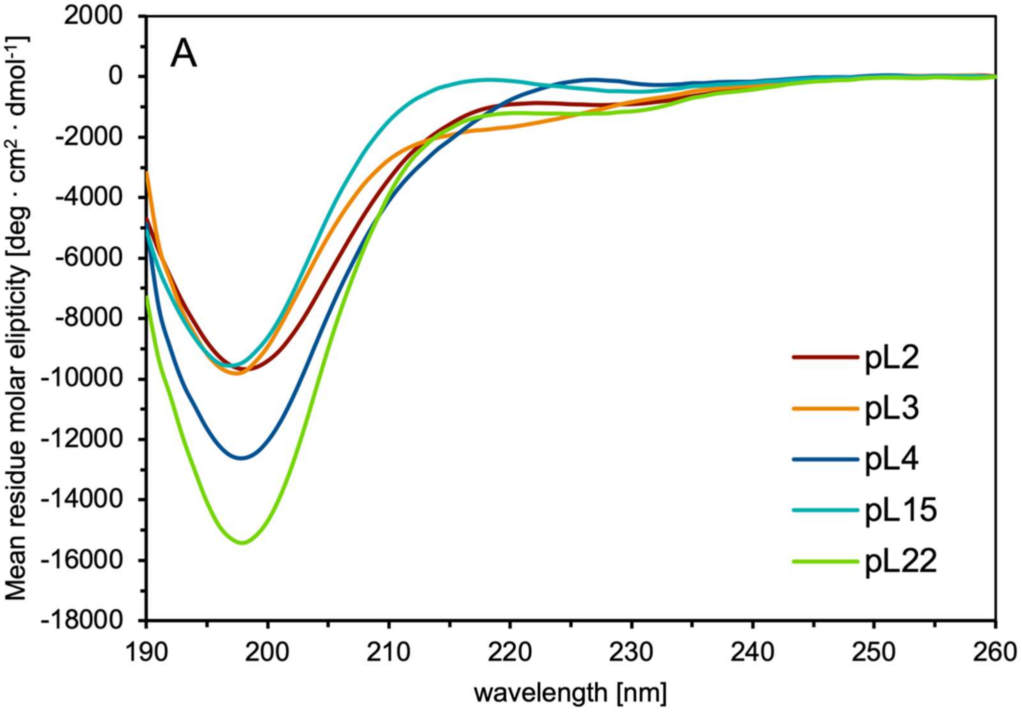

B

rPeptide	rRNA bound state		AlphaFold2	
pL2	Coil	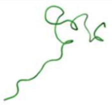	Coil	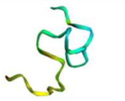
pL4	Coil	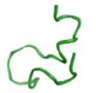	Coil	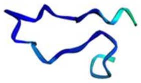
pL3	Coil	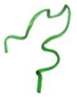	Coil	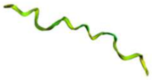
pL15	Coil	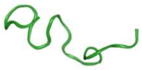	Coil	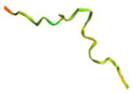
pL22	Beta-hairpin	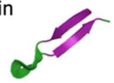	Alpha-helix region	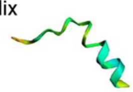

Supplementary Fig. 6. Experimental & computational secondary structure assessment of rPeptides (pL2, 3, 4, 15, 22).

(A) Far-UV CD spectra of rPeptides; spectra were collected twice for every peptide in 10 mM Tris-HCl (pH 7.5), 1 mM KCl, 1 mM $MgCl_2$, 1 mM $CaCl_2$; the average spectra are shown.

(B) Comparison between rPeptides structures as found in the rRNA bound state (PDB 4V51) and as predicted by AlphaFold2.

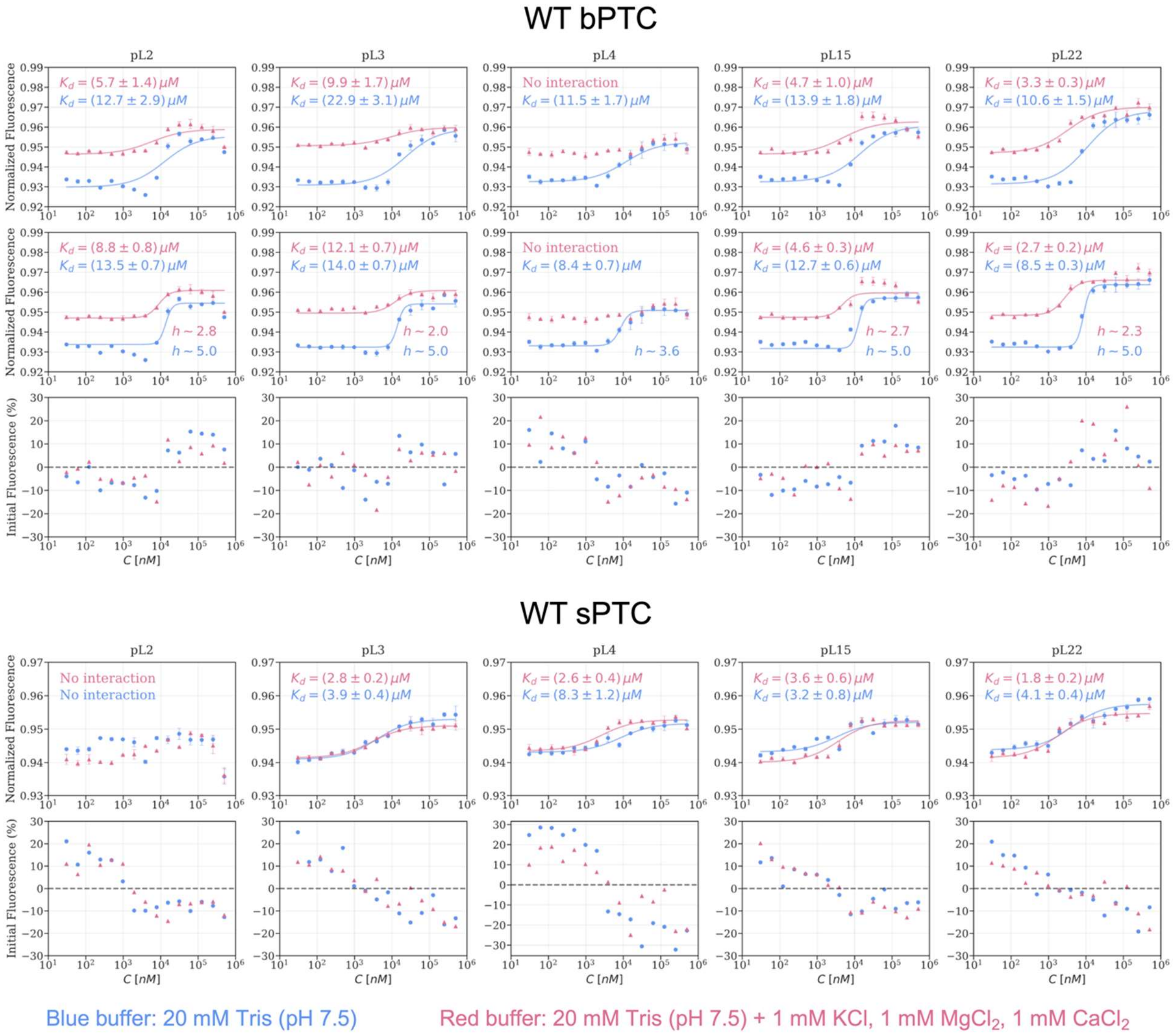

Supplementary Fig. 7. The binding curves (*upper rows*) and fluorescence traces (*lower rows*) for WT bPTC (*top panel*) and sPTC (*bottom panel*) with rPeptides pL2, 3, 4, 15, and 22 (*columns from left to right*), with (*red*) and without (*blue*) metal ions supplemented in the buffer. Solid curves in the first row of both panels represent the best fit to mass-action kinetics (*see eq. M below*). For WT bPTC, differences in the binding curves were observed in the presence vs absence of metal ions: the additional middle row in the WT bPTC panel shows the best fit to Hill functions (*see eq. H below*), where the approximate best Hill coefficients (denoted by *h*) were estimated.

Binding curves were analysed based on mass-action or Hill equations. sPTC curves are well fitted with a simple 1:1 binding reaction model:

$$F_n([L];K_d) = F_n^{(u)} + \frac{F_n^{(b)} - F_n^{(u)}}{2} \frac{[L] + [T_0] + K_d - \sqrt{([L] + [T_0] + K_d)^2 - 4[L][T_0]}}{[T_0]} \qquad (eq.M)$$

where F_n is the normalized MST signal as a function of $[L]$, the ligand molecule concentration (pL2, 3, 4, 15 or 22, x axis in the figure above), $[T_0]$ is the fixed labelled molecule concentration (here the 50 nM WT sPTC or bPTC), K_d is the dissociation constant and $F_n^{(u)}$ and $F_n^{(b)}$ are, respectively, the normalized fluorescence values of the unbounded and bounded states; binding curves were fitted against $[L]$ with free parameters $F_n^{(u)}$, $F_n^{(b)}$ and K_d.

On the contrary, the shape of the bPTC curves suggests binding with peptide:RNA stoichiometry significantly different from 1:1, which can be accounted for in the most simple terms by the cooperative model represented by Hill equation:

$$F_n([L]; K_d, h) = F_n^{(u)} + \frac{F_n^{(b)} - F_n^{(u)}}{1 + (K_d/[L])^h} \quad (eq.H)$$

where $[L]$, $F_n^{(u)}$, $F_n^{(b)}$ and K_d have the same meaning as in $(eq.M)$, while h represents the Hill exponent; binding curves were fitted against $[L]$ with free parameters $F_n^{(u)}$, $F_n^{(b)}$, K_d and h.

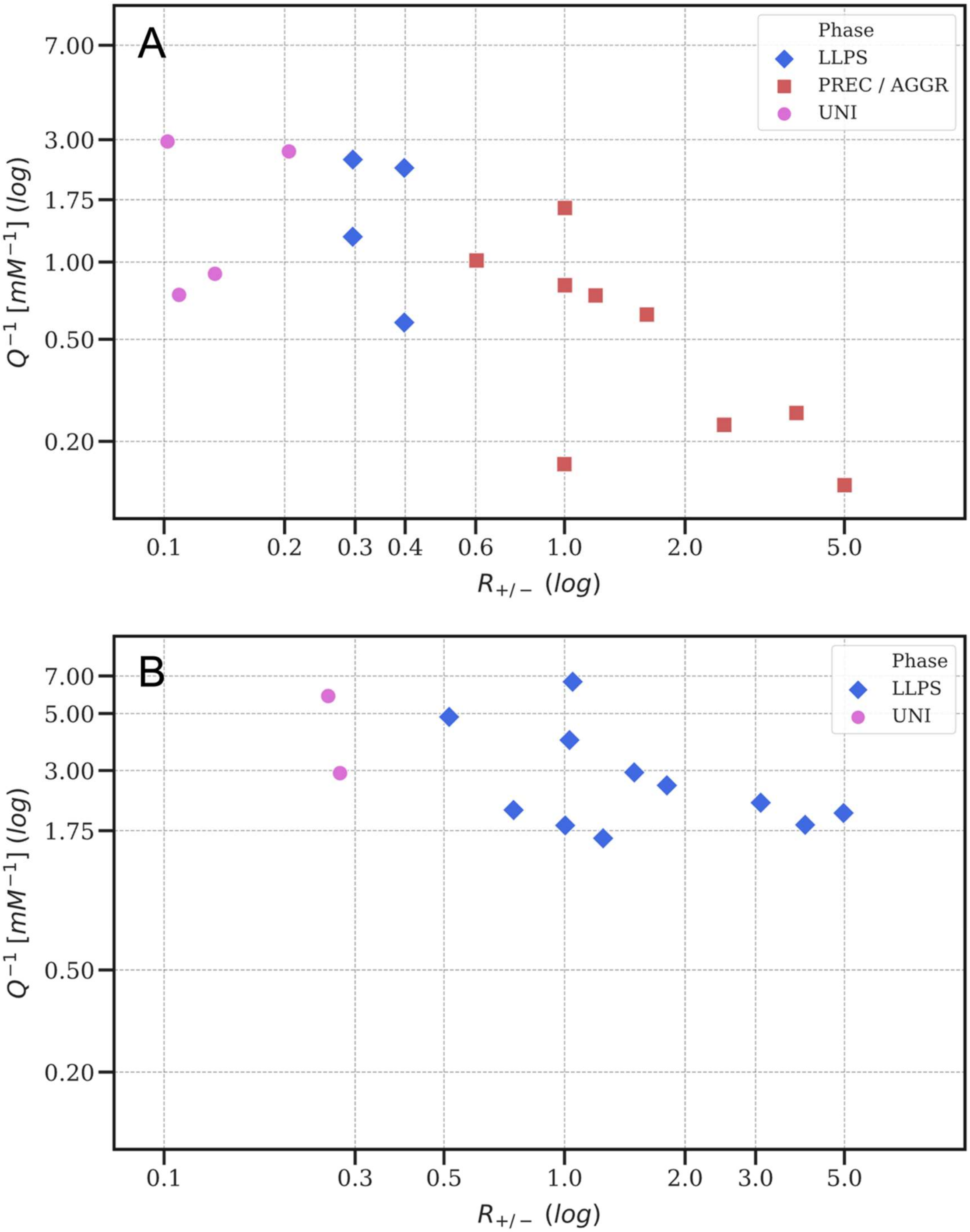

Supplementary Fig. 8. Phase diagrams of pL22 with WT bPTC (*panel A*) and WT sPTC (*panel B*) as a function of the stoichiometric charge ratio $R_{+/-}$ (x axis, see *eq. 1* in **Mat. & Meth., *coacervation***) and of the inverse of the molar concentration of charged units Q^{-1} (y axis, see *eq. 2* in ***ibidem***). Both axes are in log scale. Different colours and markers are associated to different phases, with "UNI" indicating mixed states, "PREC/AGGR" the presence of precipitates or aggregates (**Figure 4B**) and "LLPS" the formation of liquid-like droplets (**Figure 4A & F**).

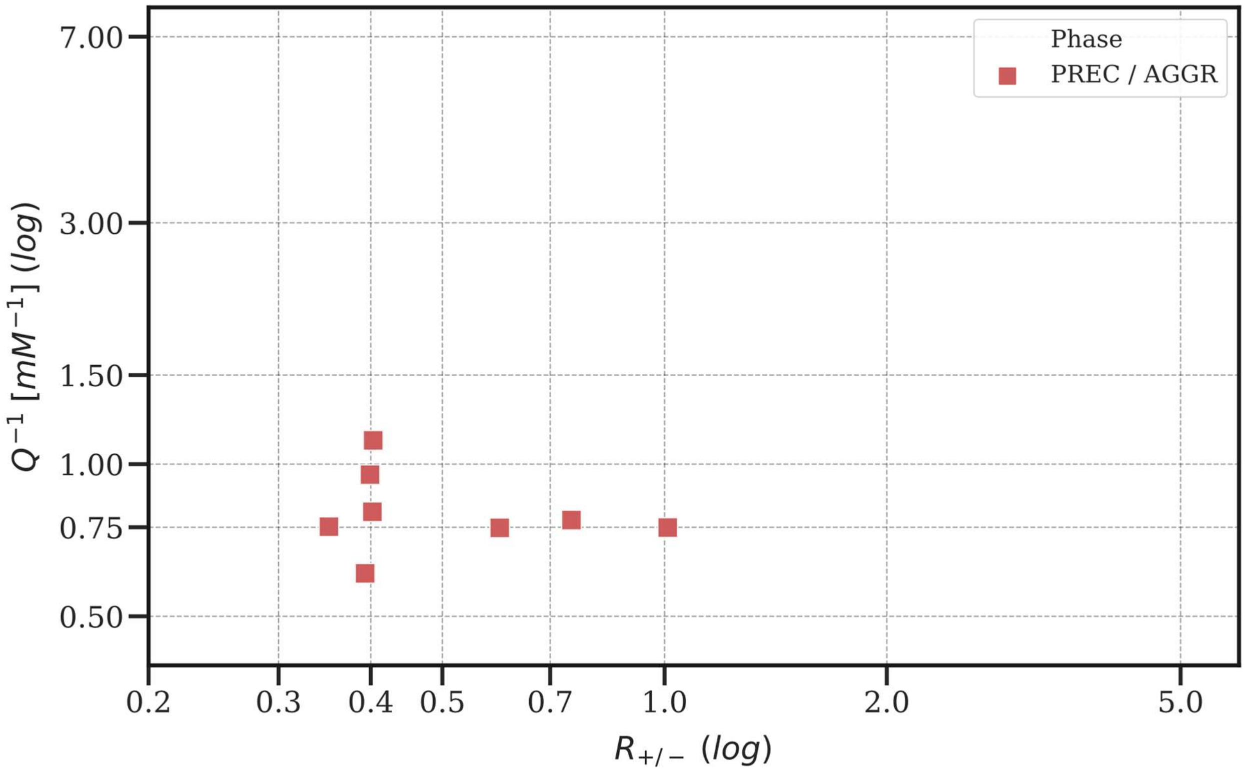

Supplementary Fig. 9. Phase diagram of mixtures of pLs (2, 3, 4, 15 and 22) with Sh bPTC as a function of the stoichiometric charge ratio $R_{+/-}$ (x axis, see *eq. 1* in **Mat. & Meth., *coacervation***) and of the inverse of the molar concentration of charged units Q^{-1} (y axis, see *eq. 2* ***ibidem***). Both axes are in log scale. Every sample examined contained some precipitates or aggregates (**Figure 4B**), thus the phase is indicated with “PREC/AGGR”, with the same colour and marker code as in **Figure 4** and in **Supplementary Fig. 8**.

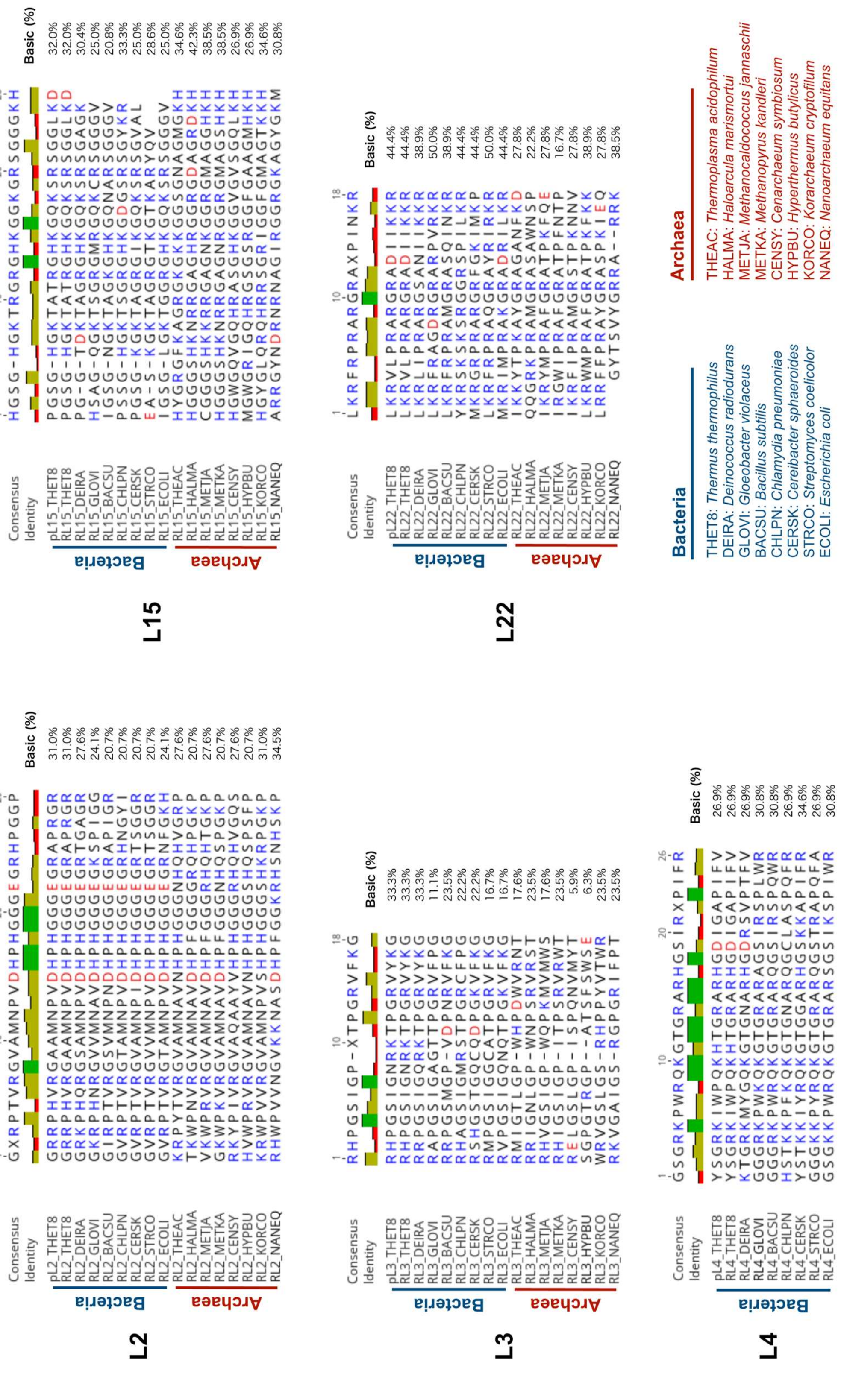
L2
L3
L4
L15
L22
Bacteria
Archaea
Consensus
Identity
Basic (%)
Bacteria
THET8: Thermus thermophilus
DEIRA: Deinococcus radiodurans
GLOVI: Gloeobacter violaceus
BACSU: Bacillus subtilis
CHLPN: Chlamydia pneumoniae
CERSK: Cereibacter sphaeroides
STRCO: Streptomyces coelicolor
ECOLI: Escherichia coli
Archaea
THEAC: Thermoplasma acidophilum
HALMA: Haloarcula marismortui
METJA: Methanocaldococcus jannaschii
METKA: Methanopyrus kandleri
CENSY: Cenarchaeum symbiosum
HYPBU: Hyperthermus butylicus
KORCO: Korarchaeum cryptofilum
NANEQ: Nanoarchaeum equitans

Supplementary Fig. 10. Multiple sequence alignment of rPeptide residues with diverse bacterial and archaeal ribosomal proteins.

Each rPeptide (pL2, pL3, pL4, pL15 and pL22) sequence was aligned with representative ribosomal protein sequences from 8 bacterial and 8 archaeal species. Multiple sequence alignment was conducted using Clustal Omega with default settings. Consensus and identity (highlighted to represent sequence conservation) for the aligned sequences are shown. Only the bacterial rL4 sequences are shown due to the lack of sequence conservation with archaeal rL4 proteins. Ratio of basic amino acids (R, K and H) is denoted next to each sequence. The full scientific names of the species are shown at the bottom right.

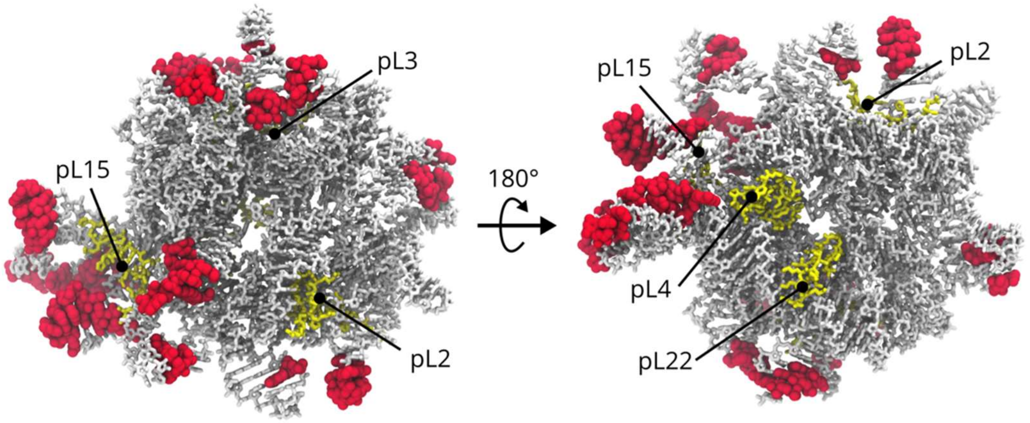

Supplementary Fig. 11. The effect of rPeptides on the structure of WT bPTC represented by RNA residues identified as the most structurally different between MD models obtained with and without rPeptides. Top 10% residues with the largest structural difference are in red, rRNA in white, rPeptides in yellow. The two viewpoints show the tRNA binding site (*left*) and the ancestral exit tunnel lined by pL4 and pL22 (*right*). For clarity, the location of pL3 is shown, although it is buried in the rRNA.

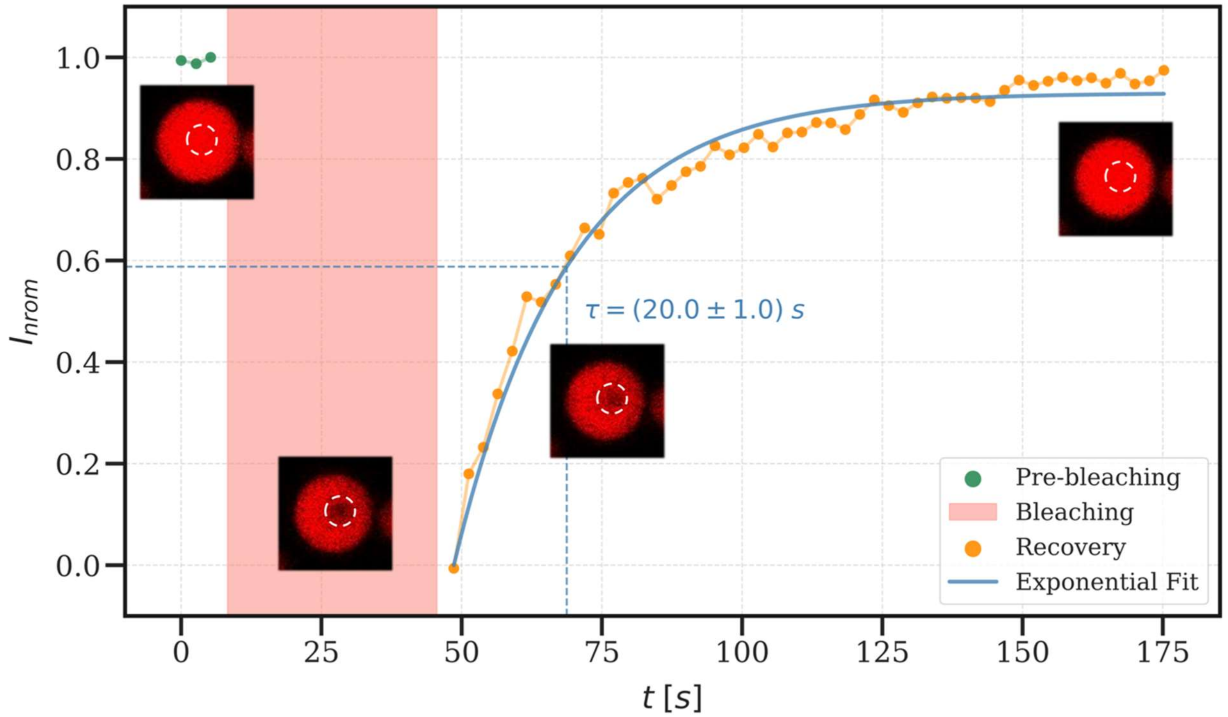

Supplementary Fig. 12. Fluorescence Recovery After Photobleaching (FRAP) experiment on the WT sPTC (2.36 μM) and pL3 (64.8 μM) mixture, with 1:200 TAMRA-labelled peptide (corresponding to R = 1.0 & Q = 0.64 mM). A small region (*dashed white circle*) inside a coacervate droplet was irradiated with a White Light Laser of a Leica Stellaris 8 DLS confocal microscope at high power for about 40 s. The plot shows that the fluorescence intensity, normalised with respect to the initial signal, fully recovers on a time scale of about 20 seconds, indicating a fluid phase.

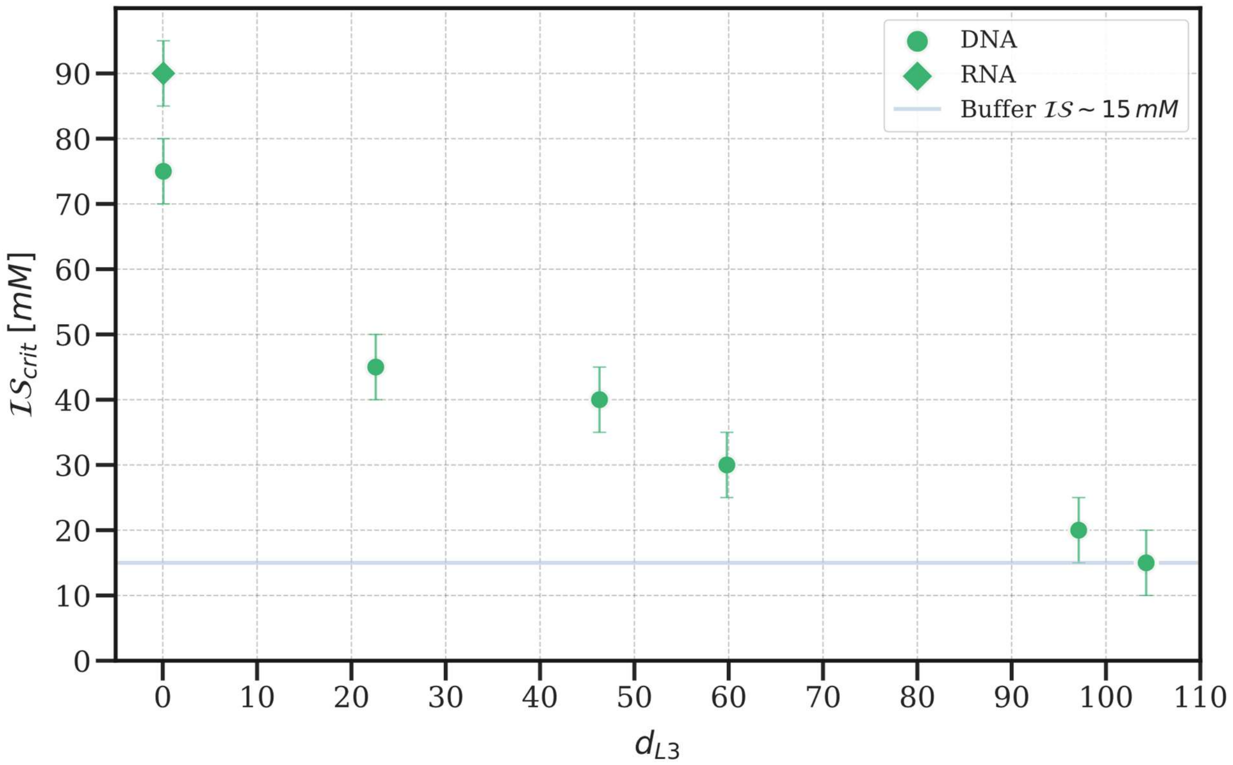

Supplementary Fig. 13. Scatter plot of the critical ionic strength (y axis) as a function of the distance (x axis) between the composition of the pL3 environment and that of several ssDNA unstructured oligomers and one RNA strand (see the legend and the "RNA" and "DNA" tags).

The critical ionic strength, measured at the same fixed DNA/RNA and peptide concentration and at ambient temperature for all the peptide-nucleic acid pairs, is defined as the highest NaCl concentration at which coacervation is still observed under a bright field microscopy setting. The distance d_{L3} is defined using the euclidean metric as:

$$d_{L3} = \sqrt{(A - A_{L3})^2 + (C - C_{L3})^2 + (G - G_{L3})^2 + (T - T_{L3})^2}$$

where A, C, G, T indicate the nucleobases percentage composition of the oligomers, while the same letters with "L3" subscript refers to the percentage composition of the pL3 *T. Thermophilus* rRNA environment, measured directly locating the 5 Angstrom closer residues to pL3 in PDB 4V51. Error bars account for a 10 mM experimental uncertainty on the measured critical ionic strength, while the grey horizontal line indicates the approximate buffer ionic strength (20 mM Tris, pH 7.6), which constitutes the experimental lower limit.

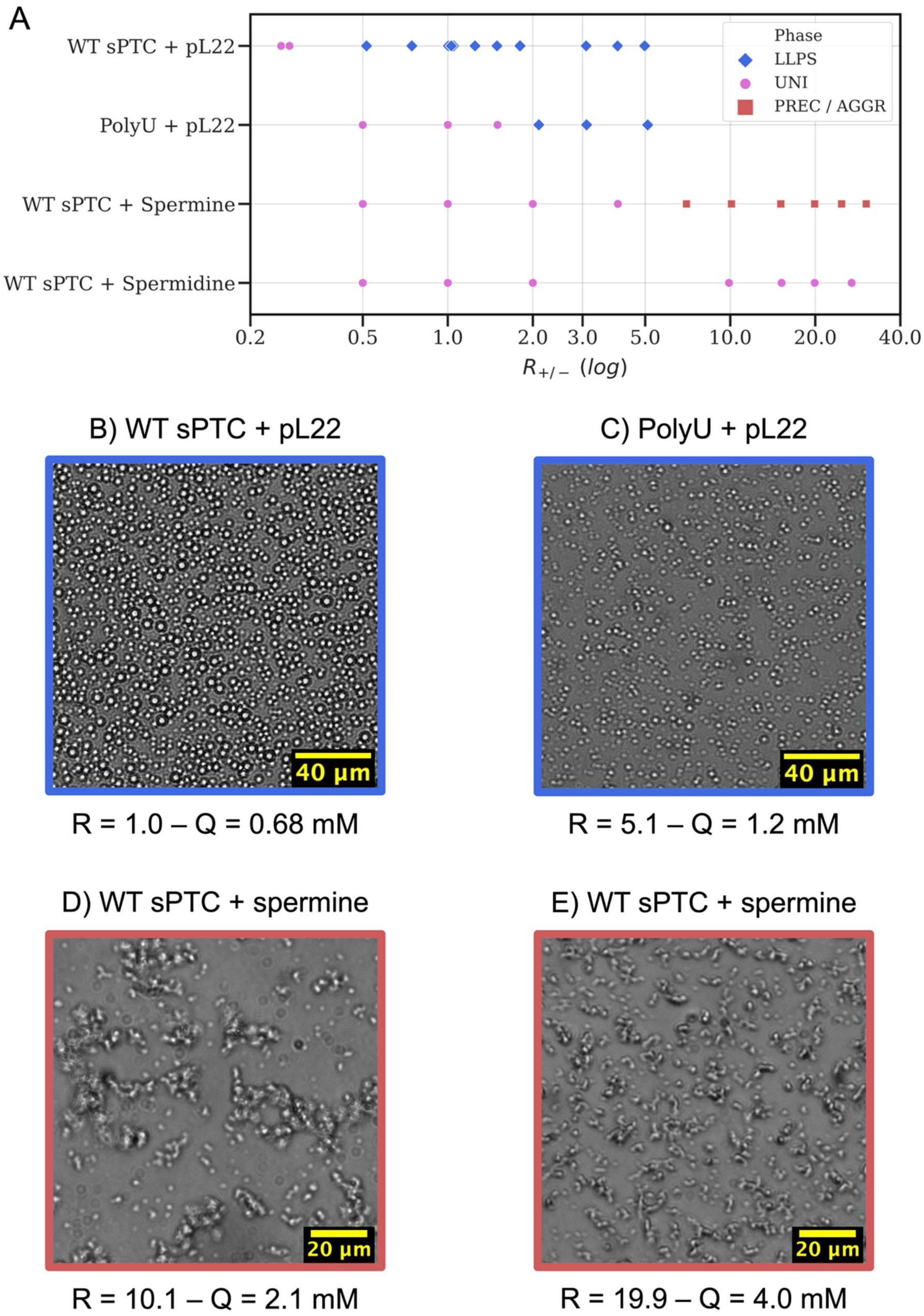
A
WT sPTC + pL22
PolyU + pL22
WT sPTC + Spermine
WT sPTC + Spermidine
Phase
LLPS
UNI
PREC / AGGR
0.2
0.5
1.0
2.0
3.0
5.0
10.0
20.0
40.0
$R_{+/-}$ (log)
B) WT sPTC + pL22
40 µm
R = 1.0 – Q = 0.68 mM
C) PolyU + pL22
40 µm
R = 5.1 – Q = 1.2 mM
D) WT sPTC + spermine
20 µm
R = 10.1 – Q = 2.1 mM
E) WT sPTC + spermine
20 µm
R = 19.9 – Q = 4.0 mM

Supplementary Fig. 14. Comparison between the phase behaviours of various RNA + polycation systems, as assed by bright field microscopy.

(A) Observed phases as a function of $R_{+/-}$ (see *eq. 1* in **Mat. & Meth., *coacervation***) for the 4 mixtures investigated: WT sPTC + pL22 (same data as in **Supplementary Fig. 8** projected on the x axis), polyU + pL22, WT sPTC + spermine (+4 charges) and WT sPTC + spermidine (+3 charges). The RNA component (either WT sPTC or PolyU) was kept at fixed concentration (close to values promoting LLPS for WT sPTC with pL22) and different $R_{+/-}$ values have been tested by changing the polycation concentration. Different colours and markers are associated to different phases, with "UNI" indicating mixed states, "PREC/AGGR" the presence of precipitates or aggregates and "LLPS" the formation of liquid-like droplets.

(B - E) Optical micrographs showing the WT sPTC + pL22 mixture at R = 1.0 and Q = 0.68 mM (B), the PolyU + pL22 mixture at R = 5.1 and Q = 1.2 mM (C), the WT sPTC + spermine mixture at R = 10.1 and Q = 2.1 mM (D) and the WT sPTC + spermine mixture at R = 19.9 and Q = 4.0 mM (E).

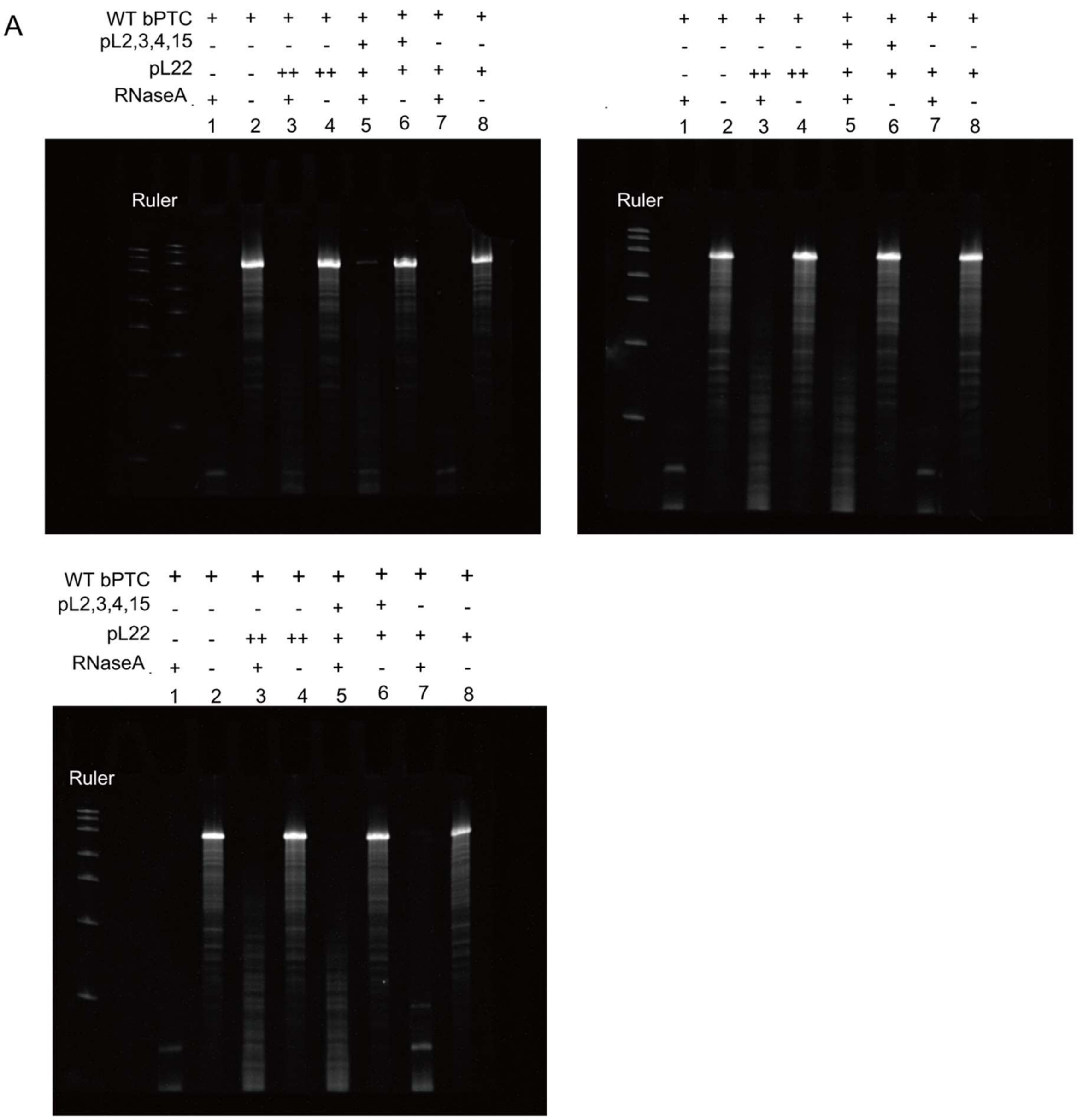
A
WT bPTC
pL2,3,4,15
pL22
RNaseA
1 2 3 4 5 6 7 8
Ruler
Ruler
WT bPTC
pL2,3,4,15
pL22
RNaseA
1 2 3 4 5 6 7 8
Ruler

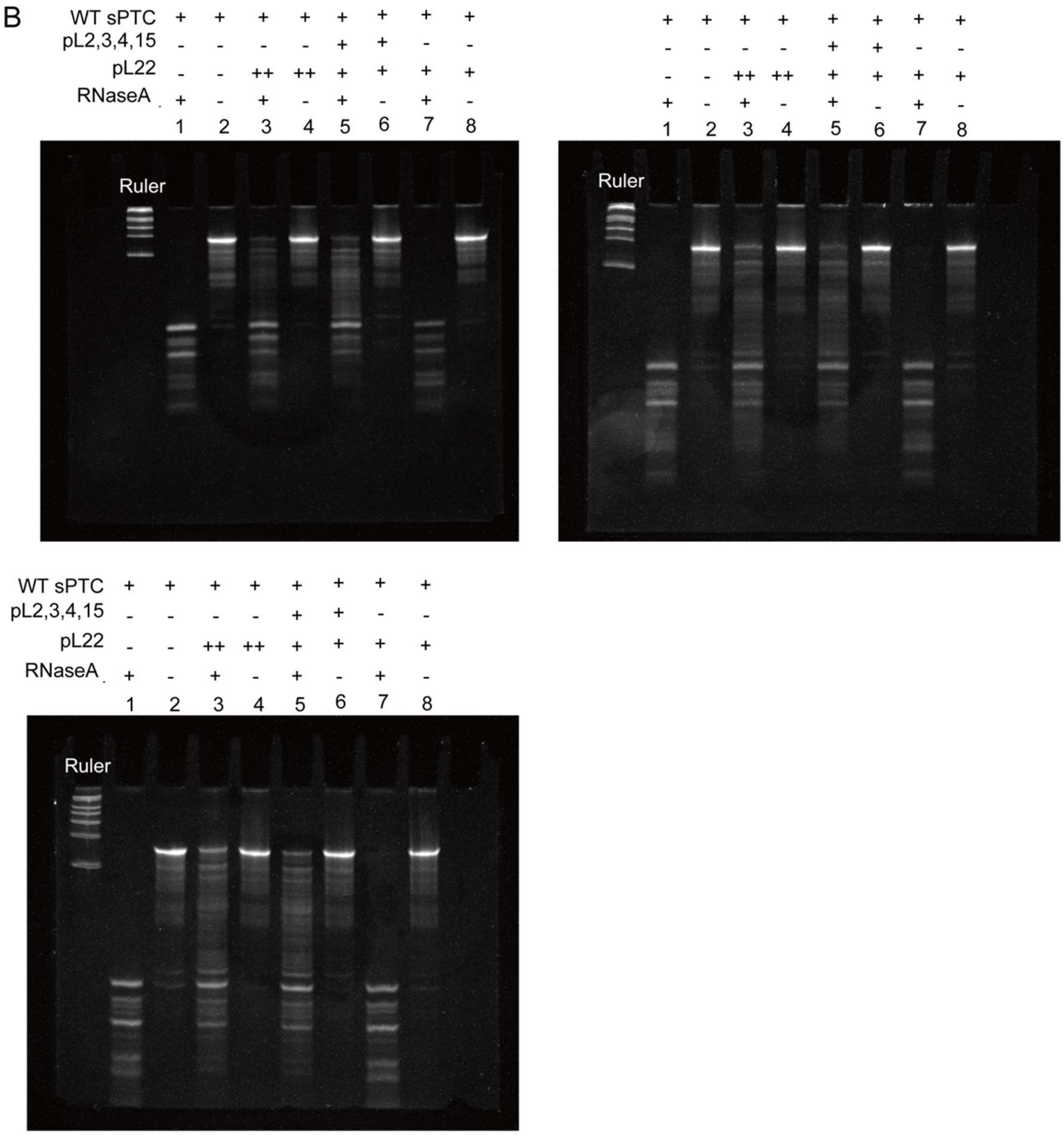

Supplementary Fig. 15. Full size Urea-gel analysis of the WT bPTC (*A*) and sPTC (*B*) RNaseA stability (see **Figure 5**). “Ruler” corresponds to RiboRuler ™ Low Range RNA ladder ready to use, Thermo Fisher Scientific.

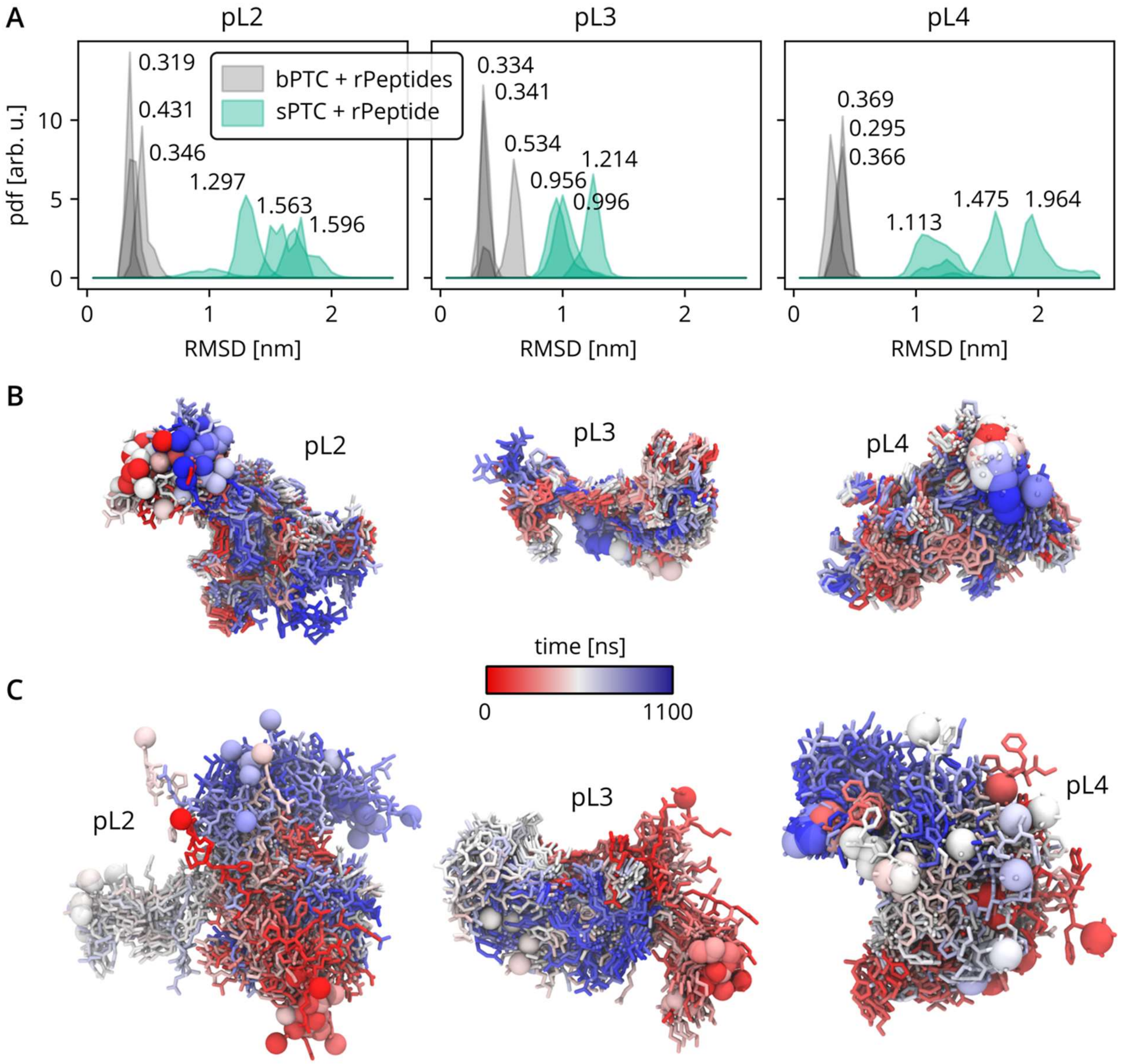

Supplementary Fig. 16. Analysis of selected rPeptides (pL2, pL3 and pL4).

(A) The root-mean-square deviation (RMSD) of rPeptides with respect to their initial position after least-square superposition of the largest common rRNA element obtained from MD simulations of WT bPTC containing all rPeptides (*grey*) or WT sPTC with one rPeptide (*green*). The mean values of the probability distribution functions (pdfs) are denoted.

(B) Representative rPeptide conformations on the bPTC surface (*not shown*), color-coded according to the simulation time from red through white to blue. The spheres represent the C-terminal $C\alpha$ atoms.

(C) Same as (*B*) but for the MD simulations of the sPTC + rPeptide.

References

1. Hsiao,C., Lenz,T.K., Peters,J.K., Fang,P.-Y., Schneider,D.M., Anderson,E.J., Preeprem,T., Bowman,J.C., O'Neill,E.B., Lie,L., *et al.* (2013) Molecular paleontology: a biochemical model of the ancestral ribosome. *Nucleic Acids Res.*, **41**, 3373–3385.

2. Bose,T., Fridkin,G., Davidovich,C., Krupkin,M., Dinger,N., Falkovich,A.H., Peleg,Y., Agmon,I., Bashan,A. and Yonath,A. (2022) Origin of life: protoribosome forms peptide bonds and links RNA and protein dominated worlds. *Nucleic Acids Res.*, **50**, 1815–1828.
